# Development and validation of machine learning-augmented algorithm for insulin sensitivity assessment in the community and primary care settings: a population-based study in China

**DOI:** 10.3389/fendo.2024.1292346

**Published:** 2024-01-25

**Authors:** Hao Zhang, Tianshu Zeng, Jiaoyue Zhang, Juan Zheng, Jie Min, Miaomiao Peng, Geng Liu, Xueyu Zhong, Ying Wang, Kangli Qiu, Shenghua Tian, Xiaohuan Liu, Hantao Huang, Marina Surmach, Ping Wang, Xiang Hu, Lulu Chen

**Affiliations:** ^1^ Department of Endocrinology, Union Hospital, Tongji Medical College, Huazhong University of Science and Technology, Wuhan, China; ^2^ Hubei Provincial Clinical Research Center for Diabetes and Metabolic Disorders, Wuhan, China; ^3^ Department of Emergency Medicine, Yichang Yiling Hospital, Yichang, China; ^4^ Department of Public Health and Health Services, Grodno State Medical University, Grodno, Belarus; ^5^ Precision Health Program, Department of Radiology, College of Human Medicine, Michigan State University, East Lansing, MI, United States

**Keywords:** insulin sensitivity assessment, machine learning, community settings, primary care settings, risk factors

## Abstract

**Objective:**

Insulin plays a central role in the regulation of energy and glucose homeostasis, and insulin resistance (IR) is widely considered as the “common soil” of a cluster of cardiometabolic disorders. Assessment of insulin sensitivity is very important in preventing and treating IR-related disease. This study aims to develop and validate machine learning (ML)-augmented algorithms for insulin sensitivity assessment in the community and primary care settings.

**Methods:**

We analyzed the data of 9358 participants over 40 years old who participated in the population-based cohort of the Hubei center of the REACTION study (Risk Evaluation of Cancers in Chinese Diabetic Individuals). Three non-ensemble algorithms and four ensemble algorithms were used to develop the models with 70 non-laboratory variables for the community and 87 (70 non-laboratory and 17 laboratory) variables for the primary care settings to screen the classifier of the state-of-the-art. The models with the best performance were further streamlined using top-ranked 5, 8, 10, 13, 15, and 20 features. Performances of these ML models were evaluated using the area under the receiver operating characteristic curve (AUROC), the area under the precision-recall curve (AUPR), and the Brier score. The Shapley additive explanation (SHAP) analysis was employed to evaluate the importance of features and interpret the models.

**Results:**

The LightGBM models developed for the community (AUROC 0.794, AUPR 0.575, Brier score 0.145) and primary care settings (AUROC 0.867, AUPR 0.705, Brier score 0.119) achieved higher performance than the models constructed by the other six algorithms. The streamlined LightGBM models for the community (AUROC 0.791, AUPR 0.563, Brier score 0.146) and primary care settings (AUROC 0.863, AUPR 0.692, Brier score 0.124) using the 20 top-ranked variables also showed excellent performance. SHAP analysis indicated that the top-ranked features included fasting plasma glucose (FPG), waist circumference (WC), body mass index (BMI), triglycerides (TG), gender, waist-to-height ratio (WHtR), the number of daughters born, resting pulse rate (RPR), etc.

**Conclusion:**

The ML models using the LightGBM algorithm are efficient to predict insulin sensitivity in the community and primary care settings accurately and might potentially become an efficient and practical tool for insulin sensitivity assessment in these settings.

## Introduction

Insulin resistance (IR)-related diseases such as obesity, type 2 diabetes, hypertension, hyperlipidemia, non-alcoholic fatty liver disease (NAFLD), and atherosclerotic cardiovascular diseases have been increasingly prevalent (1–6). A large number of patients are not able to be diagnosed and subsequently obtain management timely because they usually have long asymptomatic phases and screening tests are not always available or accessible in communities and primary care settings, which are a critical challenge in the prevention and control of these IR-related diseases and consequently increasing morbidity and mortality and imposing a heavy economic burden on patients and their health care systems globally (5, 7–10). Decreases in insulin sensitivity, which is well known as IR, are widely recognized as the common soil in the pathogenesis of these IR-related disorders (11). Fortunately, numerous studies indicate that improving insulin sensitivity by modifying its risk factors is able to prevent and/or prolong the progression of these diseases (5, 12–14). Thus, it is crucial to evaluate insulin sensitivity early and identify its risk factors in individuals potentially at-risk in the community and primary care settings.

The hyperinsulinemic-euglycemic clamp is widely considered as the gold standard for evaluating insulin sensitivity *in vivo* (15). However, it seems impractical to assess insulin sensitivity employing the hyperinsulinemic-euglycemic clamp technique for routine use in clinical practice or in the general population, since its procedure is considerably time-consuming, labor-intensive, and costly. Alternatively, the homeostasis model assessment of insulin resistance (HOMA-IR= fasting glucose (mmol/L)*fasting insulin (µU/ml)/22.5) has been gradually widely adopted to evaluate insulin sensitivity for its simplicity, low cost, and good correlation with the hyperinsulinemic-euglycemic clamp method (16, 17). However, the determination of fasting insulin is not routinely available or always accessible in the community or primary care settings. Therefore, it would be of great help to explore novel approaches which are more convenient and accessible to assess insulin sensitivity in the community and primary care settings.

Recent studies that many factors are closely related to insulin sensitivity based on logistic regression analysis and may be promising predictors in the assessment of insulin sensitivity, including the TG/HDL ratio (18), the TG/HDL ratio combined with waist circumference, gender, ALT (19), BMI (20), triglyceride glucose index (TyG) combined with obesity indicators (BMI, waist circumference, WHtR) (21), ALT/AST ratio (22), etc. That a large number of factors might affect insulin sensitivity and the influence of each factor might be different and complicated makes the prediction of insulin sensitivity challenging using traditional methods such as logistic regression analysis. It is extremely important to screen out as many as critical related factors and develop novel approaches using these potential complex predictors to accurately predict insulin sensitivity. Moreover, if these new approaches are convenient, time-saving, highly accessible, and cost-effective enough, it would be of great help for individuals in community and primary care settings to obtain diagnosis and treatment opportunely.

Machine learning (ML), as a data-driven approach, is well-known for its ability to detect complex nonlinear relationships and potential interactions between variables and outcomes and has been increasingly showing outstanding performance in predicting health-related outcomes by learning from inputted big data in clinical practice (23). ML has been used to predict the risk of hypoglycemic events in hospitalized patients (24) and heart failure in diabetic patients (25) with great performances in accuracy and efficiency (26). In this study, we aim to develop predictive models of insulin sensitivity in individuals potentially at-risk in the community and primary care settings and validate their performances, as well as screen out the vital predictors involved in these models, in the hope of providing support in the prevention and/or control of the IR-related diseases.

## Methods

### Study participants, data collection, and study design

Data for this study were collected from 10184 individuals over 40 years old who participated in the population-based cohort of the Hubei center of the REACTION study (Risk Evaluation of Cancers in Chinese Diabetic Individuals). This study was conducted in 2011 in China and has been described in detail in previous research (27). Briefly, participants received a standard questionnaire to collect information on their sociodemographic, lifestyle, exercise status, educational level, and medical history. The trained nurses used standard protocols to measure height, weight, waist circumference (WC), hip circumference (HC), systolic blood pressure (SBP), diastolic blood pressure (DBP), and resting pulse rate (RPR). The waist-to-height ratio (WHtR) and waist-to-hip ratio (WHR) were calculated as the standard method. The participants’ weight gain since the age of 20 was calculated as the difference between their current weight and self-reported weight at age 20. The weight gain ratio was calculated as the weight gain since age 20 divided by weight at age 20.

**Figure 1:**
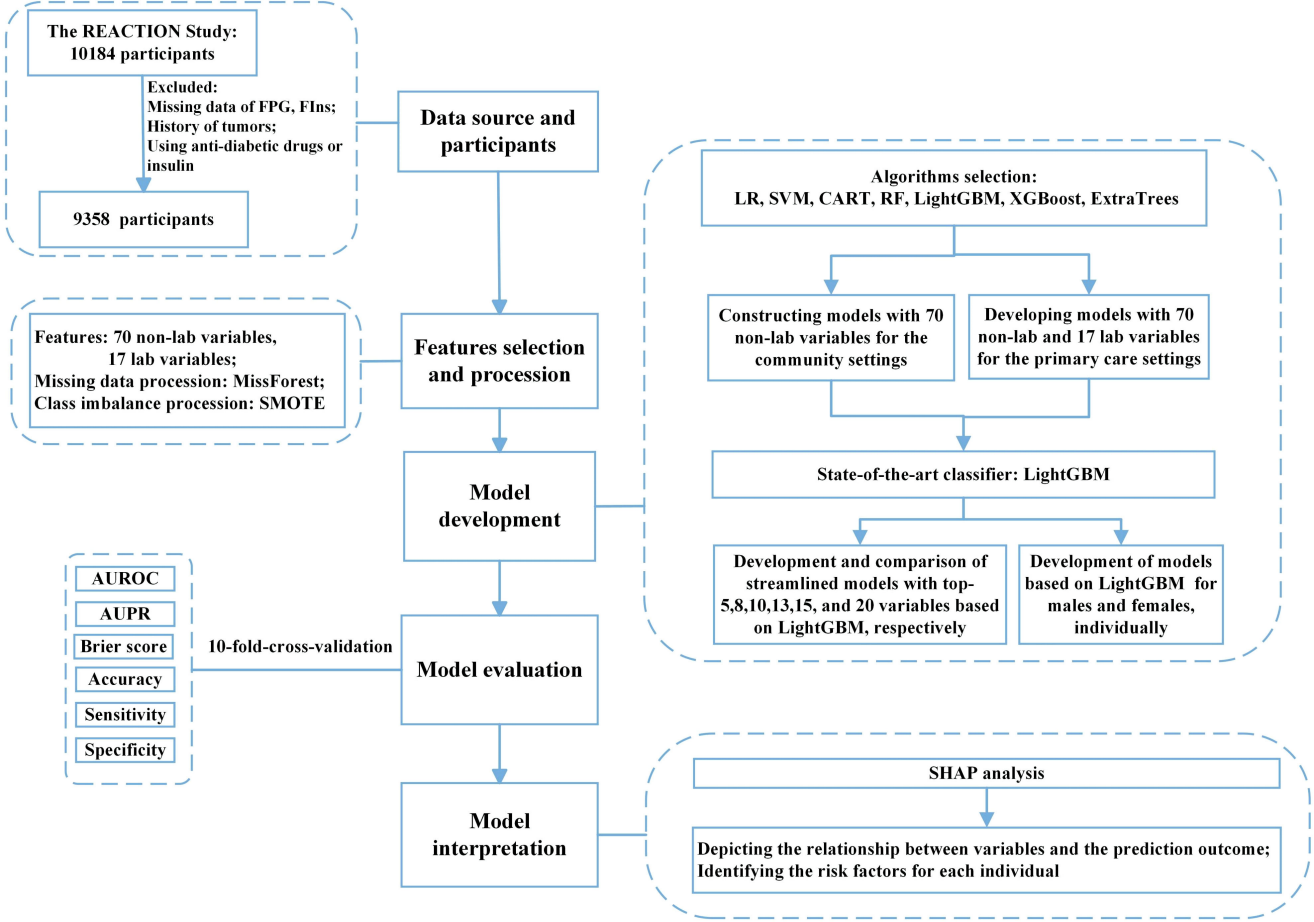
Flow diagram of the study. FPG, fasting plasma glucose; FIns, fasting serum insulin; SMOTE, synthetic minority oversampling technique; ML, machine learning; LR, logistic regression; SVM, support vector machine; CART, classification and regression tree; RF, random forest; XGBoost, eXtreme Gradient Boosting; Extra-trees, extremely randomized trees; LightGBM, light gradient boosting machine; AUROC, area under the receiver operating characteristic curve; AUPR, area under the precision-recall curve; SHAP, SHapley additive explanation.

Laboratory data were collected through fasting overnight and a 75 g oral glucose tolerance test (OGTT) was performed. Plasma glucose was measured at the local hospital using the glucose oxidase method or hexokinase method, while other blood samples were transported to the central laboratory of Ruijin Hospital for further testing. Fasting insulin (FIns) levels were measured using a chemiluminescent immunoassay. Other measurements included glycated hemoglobin (HbA1c), total cholesterol (TC), low-density lipoprotein cholesterol (LDL-C), high-density lipoprotein cholesterol (HDL-C), and triglyceride (TG) levels. Non-HDL cholesterol was defined as the difference between TC and HDL-C. The ratios of non-HDL-C to HDL-C (non-HDL-C/HDL-C), triglycerides to HDL-C (TG/HDL-C), and total cholesterol to HDL-C (TC/HDL-C) were also calculated. Participants who had been diagnosed with tumors, taken hypoglycemic agents, used insulin, or whose data on FPG, and FIns were missing were excluded and 9,358 participants were included in the analysis. The flow chart of this study is shown in **Figure 1**.

This study complies with the Declaration of Helsinki and was approved by the Ethics Committee of Tongji Medical College, Huazhong University of Science and Technology. All participants provided informed consent.

### Outcome variable

Insulin sensitivity was evaluated using the Homeostatic Model Assessment of Insulin Resistance (HOMA-IR) index: HOMA-IR = fasting glucose (mmol/L)*fasting insulin (µU/ml)/22.5 (16, 28). Participants with HOMA-IR values greater than the third quartile (HOMA-IR≥2.26) of the study population were considered to be low insulin sensitivity (low IS, labeled as 1), while those with HOMA-IR values less than 2.26 were considered to be high insulin sensitivity (high IS, labeled as 0) as described previously (29, 30).

### Feature processing and algorithm selection

A total of 87 variables, including 70 non-laboratory and 17 laboratory variables, were included in the analysis. Missing values of variables were imputed using the MissForest method (31). The Synthetic Minority Oversampling Technique (SMOTE) was utilized to address the problem of class imbalance (low IS and high IS) to obtain better performances of the predictive models (32). Three non-ensemble algorithms [Logistic Regression (LR), Classification and Regression Tree (CART), and Support Vector Machine (SVM)] and four ensemble algorithms [Random Forest (RF), eXtreme Gradient Boosting (XGBoost), Extremely randomized trees (ExtraTrees), and Light Gradient Boosting Machine (LightGBM)] were employed to develop the predictive models of insulin sensitivity assessment in the community and primary care settings.

### Model development in community and primary care settings

In this study, we developed predictive models in the settings of community and primary care and evaluated their performances. For internal validation, 10-fold stratified cross-validation was used in the present study, in which the dataset was randomly divided into ten sets, with nine used for training and one for validation to reduce variance in prediction errors and prevent overfitting (33). The training set in models for the general population initially held 8423 instances, while the validation set contained 935 before applying SMOTE. After the SMOTE algorithm processing, the training set achieved class balance with 12626 instances, and the validation set remained 935. Given that those non-laboratory indexes were more accessible in communities, the non-laboratory variables were utilized to build the predictive models of insulin sensitivity assessment in community settings. Laboratory indicators which are usually available in primary care providers were further incorporated to create the insulin sensitivity predictive models in the setting of primary care with as great performance as possible. The models performed a binary classification task, predicting whether the subject falls into class 0 (high IS) or class 1 (low IS). Six streamlined models were developed using 5, 8, 10, 13, 15, and 20 features of top-ranked importance among all the variables of the state-of-the-art model (LightGBM) to simplify the predictive models for practice. These streamlined models with the best performances and minimum number of variables were adopted as the predictive models for insulin sensitivity assessment in the community and primary care settings. Moreover, GridSearchCV was employed to tune hyperparameters and improve model prediction performance and the detailed information about the hyperparameters used for each machine learning algorithm was shown in **Supplementary Tables 1**, **2**. These hyperparameters were not varied during the study to ensure the reproducibility of the research.

### Evaluation of model performance in community and primary care settings

The models were evaluated using 10-fold stratified cross-validation and the experiments were repeated for ten times, generating metrics (e.g., AUROC) in each time, which were averaged to evaluate the model performances. The discrimination performance of the model was evaluated using AUROC and AUPR (34, 35). The calibration of the model was evaluated using the Brier score (36). We also used sensitivity, specificity, and accuracy to evaluate the predictive capacity of the model (35). True Positive (TP) indicated the number of true positives, False Positive (FP) indicated the number of false positives, True Negative (TN) indicated the number of true negatives, and False Negative (FN) indicated the number of false negatives. Sensitivity was defined as TP/(TP + FN), also known as the true positive rate, which was the percentage of actual positives that were correctly identified by the model and reflected the ability to identify patients. Specificity was defined as TN/(TN + FP), also known as the true negative rate, which was the percentage of actual negatives that were correctly judged as negatives by the model, reflecting the ability to identify non-patients. Accuracy was defined as (TP+TN)/(TP+TN+FP+FN), which indicated the ratio of the number of correct samples predicted by the model to the total sample size. The Receiver Operating Characteristics curve, Precision-Recall curve, and Calibration curve were adopted to visualize the model performance. The overall performance was evaluated by averaging the performance in each experiment of the 10-fold stratified cross-validation mentioned.

### Model development and performance evaluation in sex-specific populations

In view that differences may occur in the assessment of insulin sensitivity between males and females, we tried to construct the predictive models of insulin sensitivity assessment in the settings of community and primary care for male and female populations and evaluated their performances using 10-fold stratified cross-validation and applying SMOTE as mentioned above. In male, the training and validation sets comprised 3012 and 334 cases respectively before the processing of SMOTE algorithm, and included 4508 and 334 cases individually after applying SMOTE. In female, the training and validation sets involved 5411 and 601 cases separately before using SMOTE, and contained 8120 and 601 cases respectively after the application of SMOTE.

### Feature importance evaluation and model interpretation

The contribution to the state-of-the-art model (LightGBM) of the features was evaluated using the SHapley Additive explanation (SHAP) analysis (37). SHAP summary plots were employed to summarize the impact of each feature in the model, while SHAP dependence plots were adopted to show the correlation between the features and the predicted outcome. A positive SHAP value indicated that the feature has a positive effect on the model output, a negative SHAP value indicated a negative impact, and the higher absolute SHAP value represented a greater impact. Two participants were selected at random as examples to demonstrate the feature importance evaluation and model interpretation.

### Statistical analysis

Data for continuous variables were presented as means ± standard deviations(normally distributed) or median ± interquartile range (nonnormally distributed), while data for categorical variables were shown as frequency (percentage). Statistical analysis was performed using SPSS software (v.26.0 IBM) software, with the t-test, the Mann-Whitney U test, and χ^2^ test applied to test for differences between groups for normally distributed continuous variables, nonnormally distributed variables, and categorical variables, respectively. A two-sided *P* value <0.05 was considered statistically significant. ML models were developed using the scikit-learn package (0.24.1) in Python 3.8. The SHAP analysis was conducted using the implementation available at http://github.com/slundberg/shap.

## Results

### Basic characteristics of the participants

Among the 9,358 participants enrolled, there were 2,344 (25%) subjects with HOMA-IR greater than 2.26 and thus identified as low IS. The demographic and biochemical characteristics of the participants were shown in **Table 1**. Compared to high IS group, people with low IS had significantly higher levels of WC, HC, BMI, WHR, WHtR, weight gain since age 20, weight gain ratio since age 20, SBP, DBP, RPR, FPG, FIns, P2hPG, HbA1c, creatinine, LDL-C, TG, TC, TG/HDL-C, ALT, ALT/AST, and γ-GGT (*P* values <0.05). Height, HDL-C/LDL-C, HDL-C, and the proportion of smoking and drinking were significantly lower (*P* values <0.05). No significant difference was observed in age, AST, and education levels between the two groups *(P* values > 0.05).

**Table 1:** Characteristics of participants.

	High IS (HOMA-IR<2.26)	Low IS (HOMA-IR≥2.26)	*P*-value
Total	7014	2344	
Age(years)^a^	55.00(13.00)	55.00(12.00)	0.925
Females,n(%)^c^	4261(60.75)	1751(74.70)	0.000
Education,n(%)^c^			
≤Primary school	3473(49.52)	1154(49.23)	
≥Middle school	3541(50.48)	1190(50.77)	0.812
Snore,n(%)^c^	898(12.80)	421(17.96)	0.000
Medical history, n(%)^c^			
CVD	109(1.55)	59(2.52)	0.002
Hypertension	440(6.27)	287(12.24)	0.000
Dyslipidemia	59(0.84)	48(2.05)	0.000
Diabetes mellitus	90(1.28)	99(4.22)	0.000
Smoking status			
Current, n(%)^c^	1477(21.06)	249(10.62)	0.000
Former, n(%)^c^	1720(24.52)	330(14.08)	0.000
Drinking status			
Current, n(%)^c^	993(14.16)	154(6.57)	0.000
Former, n(%)^c^	1117(15.93)	202(8.62)	0.000
Weight(kg)^a^	55.00(11.00)	61.50(13.50)	0.000
Height(cm)^a^	156.32(10.00)	155.50 (9.50)	0.000
SBP (mmHg)^b^	136.54(21.10)	141.91 (21.24)	0.000
DBP (mmHg)^b^	78.86(12.12)	82.41(12.07)	0.000
RPR (bpm)^b^	80.31(11.96)	83.67(12.15)	0.000
WC (cm)^b^	76.34(8.22)	84.30(8.64)	0.000
HC (cm)^a^	89.00(8.00)	94.00(9.00)	0.000
BMI (kg/m^2^)^b^	22.65(2.86)	25.52(3.23)	0.000
Weight gain since age 20 (kg)^b^	2.22(8.22)	8.78(9.45)	0.000
Weight gain ratio since age 20^b^	0.05(0.16)	0.18(0.19)	0.000
WHR^b^	0.85(0.06)	0.89(0.07)	0.000
WHtR^b^	0.49(0.05)	0.54(0.05)	0.000
FPG (mmol/L)^a^	5.59(0.72)	6.12(1.13)	0.000
P2hPG (mmol/L)^a^	6.70(2.25)	7.76 (3.55)	0.000
HbA1c (%)^a^	5.50 (0.60)	5.70(0.70)	0.000
FIns (μU/ml)^a^	5.00(2.70)	10.60(3.80)	0.000
Creatinine (mmol/L)^a^	65.40(12.20)	66.30(12.70)	0.000
HDL-C (mmol/)^b^	1.74(0.41)	1.53(0.36)	0.000
LDL-C (mmol/L)^b^	2.93(0.81)	3.15(0.82)	0.000
TC (mmol/L)^b^	5.31(0.97)	5.49(1.00)	0.000
TG (mmol/L)^a^	1.09(0.72)	1.63(1.15)	0.000
TG/HDL-C^a^	0.65(0.54)	1.08(1.01)	0.000
ALT(u/L)^a^	16.00(9.00)	19.00(12.00)	0.000
AST(u/L)^a^	24.00(8.00)	24.00(9.00)	0.129
γ-GGT(u/L)^a^	18.00(14.00)	25.00(22.00)	0.000
ALT/AST^a^	0.65(0.28)	0.78(0.35)	0.000

IR, insulin resistance; IS, insulin sensitivity; CVD, cardiovascular disease; SBP, systolic blood pressure; DBP, diastolic blood pressure; RPR, resting pulse rate; WC, waist circumference; HC, hip circumference; BMI, body mass index; WHR, waist-to-hip ratio; WHtR, waist-to-height ratio; FPG, fasting plasma glucose; P2hPG, two-hour post-load plasma glucose; HbA1c, glycosylated hemoglobin A1c; FIns, fasting insulin; HDL-C, high-density lipoprotein cholesterol; LDL-C, low-density lipoprotein cholesterol; TC, total cholesterol; TG, triglyceride; ALT, alanine aminotransferase; AST, aspartate aminotransferase; γ-GGT, γ-glutamyl transpeptidase.

a Data were presented as median (interquartile).

b Data were presented as mean (standard deviation).

c Data were displayed as frequencies (percentages).

### Evaluation performance of models developed for the community and primary care settings

**Figure 2:**
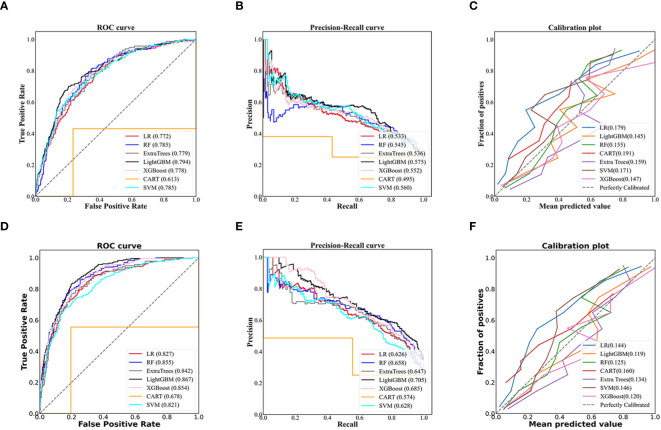
Evaluation of predictive models based on seven algorithms (LR, RF, Extra trees, LightGBM, XGBoost, CART, SVM). The receiver operating characteristic curve, precision-recall curve, and calibration curve of the models using 70 non-laboratory variables for the community settings **(A–C)** and the models employing 70 non-laboratory and 17 laboratory variables for the primary care settings **(D–F)**, and LightGBM demonstrated the best performance among the seven algorithms in the models for community and primary care setting, individually. LR, Logistic Regression; RF, Random Forest; ExtraTrees, Extremely randomized trees; LightGBM, Light Gradient Boosting Machine; XGBoost, eXtreme Gradient Boosting; CART, Classification and Regression Tree; SVM, Support Vector Machine.

The LightGBM model for community setting achieved the highest performance among the machine learning models developed by three non-ensemble algorithms and four ensemble algorithms mentioned above, with an AUROC of 0.794, AUPR of 0.575, Brier score of 0.145, accuracy of 0.785, specificity of 0.900, and sensitivity of 0.441, respectively (**Figures 2A–C**; **Table 2**).

**Table 2:** Performances of ML models using all features for the community and primary care settings.

	LR	SVM	RF	ExtraTrees	LightGBM	XGBoost	CART
Models for Community Settings							
Accuracy	0.701	0.708	0.779	0.777	0.785	0.781	0.697
Sensitivity	0.715	0.730	0.469	0.413	0.441	0.439	0.454
Specificity	0.697	0.701	0.883	0.899	0.900	0.895	0.778
Models for Primary Care Settings							
Accuracy	0.754	0.739	0.811	0.814	0.822	0.820	0.734
Sensitivity	0.742	0.746	0.604	0.534	0.578	0.575	0.547
Specificity	0.758	0.736	0.881	0.907	0.904	0.902	0.796

ML, machine learning; LR, Logistic Regression; SVM, Support Vector Machine; RF, Random Forest; ExtraTrees, Extremely randomized trees; LightGBM, Light Gradient Boosting Machine; XGBoost, eXtreme Gradient Boosting; CART, Classification and Regression Tree.

Likewise, the LightGBM model for primary care setting demonstrated the best performance in models created by the seven algorithms mentioned above, with an AUROC of 0.867, AUPR of 0.705, Brier score of 0.119, accuracy of 0.822, specificity of 0.904, and sensitivity of 0.578, individually (**Figures 2D–F**; **Table 2**).

### Feature importance evaluation and model interpretation in LightGBM model developed for the community and primary care settings

**Figure 3:**
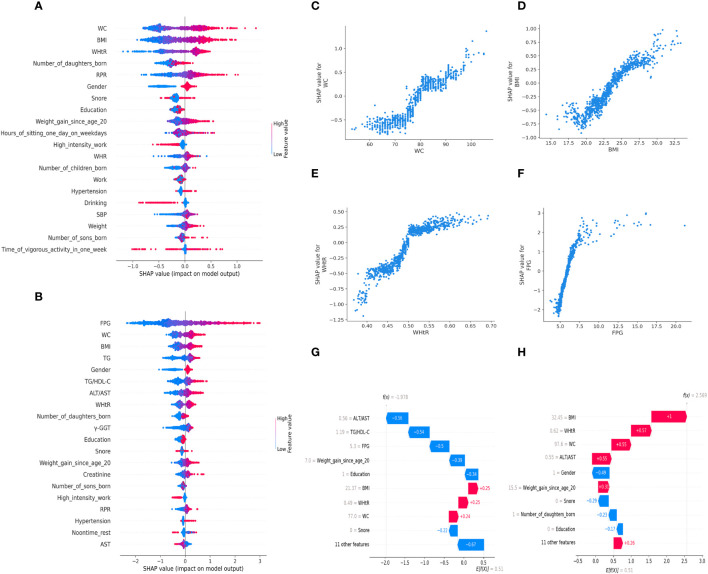
SHAP values-based interpretation of the LightGBM models in the general population. The contribution of the top-20 features is arranged in descending order of LightGBM model developed for the community and primary care settings. Red points indicate higher feature values and blue points indicate lower values **(A, B)**. The relationship between SHAP values and the levels of the top-ranked 3 features in the models for the community settings (WC, BMI, WHtR) **(C–E)** and the primary care settings (WC, BMI, FPG) **(C, D, F)**. Personalized prediction of low insulin sensitivity for two participants randomly from the validation set of the data. The color red indicates positive SHAP values, which increase the predicted value, while blue indicated negative SHAP values, which decrease the predicted value. Each arrow represents how a specific feature increases (red) or decreases (blue) the participant’s risk for low IS. If f(x) is greater than zero, the participant has a higher risk of low insulin sensitivity relative to the background population **(G, H)**. “Gender=1” means “female”, “Snore=0” means “the participant never snored”, and “Education=0” means “education levels were less than six years”. WC, waist circumference; BMI, body mass index; WHtR, waist-to-height ratio; RPR, resting pulse rate; WHR, waist-to-hip ratio; SBP, systolic blood pressure; FPG, fasting plasma glucose; TG, triglyceride; HDL-C, high-density lipoprotein cholesterol; ALT, alanine aminotransferase; AST, aspartate aminotransferase; γ-GGT, γ-glutamyl transpeptidase.

The SHAP summary plots showed that the top-20 most important features were WC, BMI, WHtR, number of daughters born, RPR, et al. in models for community settings (**Figure 3A**), and were FPG, WC, BMI, TG, Gender, et al. in models for the primary care settings (**Figure 3B**).

The SHAP dependence plots revealed that the points with SHAP values of zero, were 80cm in WC, 24.4kg/m^2^ in BMI, 0.50 in WHtR, 6.0mmol/L in FPG, etc. (**Figures 3C–F**).

Our results indicated that the variables, “ALT/AST=0.56”, “TG/HDL-C=1.19”, and “FPG=5.3 mmol/L” were the primary drivers of the prediction towards “high IS” in Participant 1 who was labeled as “0” (high IS) in the test set (**Figure 3G**), and the variables such as “BMI=32.45 kg/m^2^”, “WHtR=0.62”, and “WC=97.6 cm” were the key risk factors that prompted the model to classify Participant 2 as low IS, who was labeled as “1” (low IS) in the test set (**Figure 3H**).

### Evaluation performance of streamlined models using the top ranked-5, 8, 10, 13, 15, and 20 variables in the community and primary care settings

**Figure 4:**
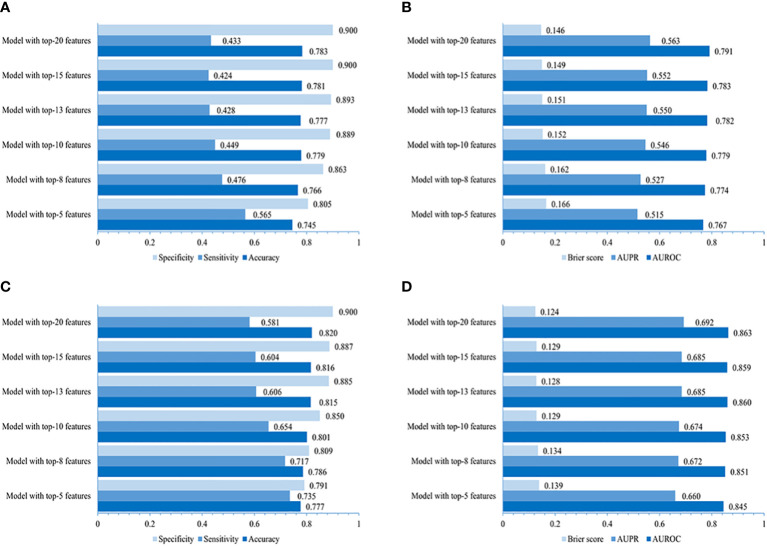
Performances of simplified LightGBM models. The value of accuracy, sensitivity, specificity, AUROC, AUPR, and Brier score of models using 5, 8, 10, 13, 15, and 20 top-ranked features in the community **(A, B)** and primary care settings **(C, D)**.

The performance of the LightGBM model developed for the community is higher in that using the top-20 non-laboratory features (AUROC of 0.791, AUPR of 0.563, the lowest Brier score of 0.146, accuracy of 0.783, sensitivity of 0.433 and specificity of 0.900, individually), compared to those using the top 5, 8, 10, 13 or 15 variables (**Figures 4A, B**), which was very close to that of LightGBM model using all 70 non-laboratory variables (AUROC 0.794, AUPR 0.575, Brier score 0.145, accuracy 0.785, specificity 0.900, and sensitivity 0.441, respectively).

The LightGBM model created for primary care adopting the top-20 variables (including laboratory variables) performed better (AUROC 0.863, AUPR 0.692, Brier score 0.124, accuracy 0.820, specificity 0.900, and sensitivity 0.581, individually) when compared to those using the top 5, 8, 10, 13, or 15 features (**Figures 4C, D**), indicating a performance similar to that of the LightGBM model using all 87 variables (AUROC 0.867, AUPR 0.705, Brier score 0.119, accuracy 0.822, specificity 0.904, and sensitivity 0.578).

### Model development for males/females and analysis of the importance of features

**Figure 5:**
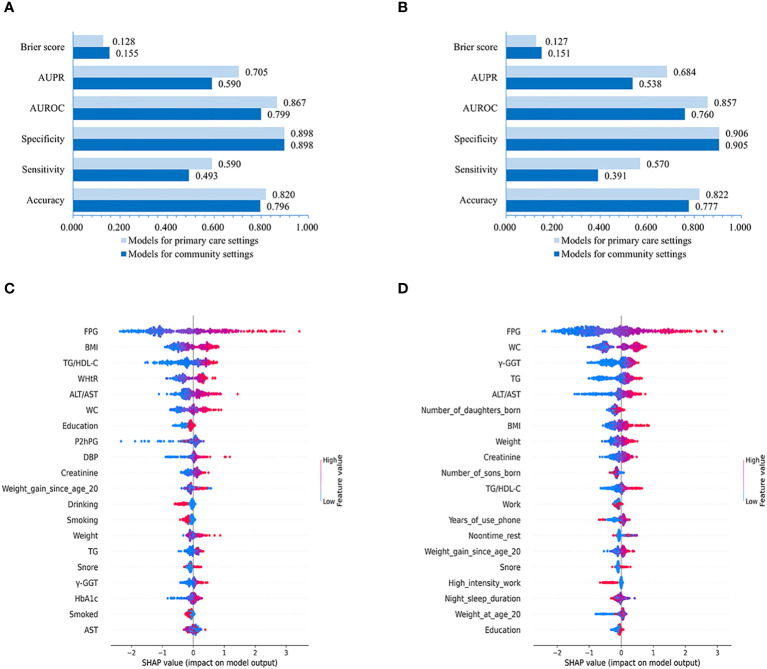
Performances of LightGBM model for males and females and SHAP values-based interpretation of the models. Evaluation of LightGBM model for males **(A)** and females **(B)**. The contribution of the 20 top-ranked features are arranged in descending order of LightGBM model developed for males and females **(C, D)**. The vertical coordinate (y-axis) shows the features in decreasing order of importance, while the horizontal coordinate (x-axis) displays the average absolute SHAP value of each feature. Red points indicate higher feature values, while blue points represent lower values.

Our results evaluating the performance of LightGBM models created for male and female populations separately demonstrated that the models for males achieved slightly higher or non-significantly inferior AUROC (0.799 vs. 0.760 with non-laboratory and 0.867 vs. 0.857 with laboratory features), AUPR (0.590 vs. 0.538 using non-laboratory and 0.705 vs. 0.684 using laboratory variables), Brier score (0.155 vs. 0.151 using non-laboratory and 0.128 vs. 0.127 using laboratory variables), sensitivity (0.493 vs. 0.391 using non-laboratory and 0.590 vs. 0.570 using laboratory features), accuracy (0.796 vs. 0.777 using non-laboratory and 0.820 vs. 0.822 using laboratory features) and specificity (0.898 vs. 0.905 with non-laboratory and 0.898 vs. 0.906 with laboratory features) compared to those for females (**Figures 5A, B**).

Further feature importance analysis revealed that in the LightGBM models with all 86 variables, the top-10 ranked features for males were FPG, BMI, TG/HDL-C, WHtR, ALT/AST, WC, Education, P2hPG, DBP, and Creatinine (**Figure 5C**), while the top-10 ranked contributors for females were FPG, WC, γ-GGT, TG, ALT/AST, number of daughters born, BMI, Weight, Creatinine, and number of sons born (**Figure 5D**).

## Discussion

In the present study, our findings revealed that the model developed using LightGBM for insulin sensitivity assessment in the community and primary care settings showed superior performance to those created by LR, RF, SVM, ExtraTrees, XGBoost, and CART. Additionally, the performance was great for the prediction of insulin sensitivity in the models developed using non-laboratory variables for the community and the models established using non-laboratory combined with laboratory features for primary care, although the performance of the latter seemed to outperform the former. Moreover, the streamlined LightGBM model for the insulin sensitivity estimation using the 20 top-ranked variables had a similar performance to the model created with all features. Noteworthy, the performance of the models developed for men using LightGBM was better than that developed for women.

In our study, the LightGBM model exhibited superior performance in terms of accuracy, specificity, AUROC, AUPR, and Brier score compared to LR, suggesting that the LightGBM model demonstrates higher predictive accuracy and calibration. Lately, it has been reported that LightGBM is considered an advanced algorithm for developing gestational diabetes risk predictive models using electronic health records (38), which is consistent with our results. LightGBM is one of the most recent successful research findings among ML approaches based on Gradient Boosting Decision Tree (GBDT) implementation with Gradient-based One-Side Sampling and Exclusive Feature Bundling, which largely reduces the training and computation cost, and speeds up the training process of conventional GBDT by up to over 20 times while achieving almost the same accuracy. It has been used for many different types of data mining tasks such as classification, regression, and ranking (39). Recent studies indicate that GBDT-based models, such as XGBoost, outperform logistic regression (LR), K-Nearest Neighbor (KNN), decision tree (DT), support vector machines (SVM), artificial neural networks (ANN) and deep neural network (DNN), in predicting insulin resistance, supporting the superior predictive accuracy of GBDT-based models (40, 41), which might be an important explanation of our findings that the LightGBM model is a better choice in developing models for insulin sensitivity in the community and primary care settings.

Our results indicated that the performance was great for the insulin sensitivity assessment in the models developed using non-laboratory features easily obtained for the community and the models established using non-laboratory combined with laboratory variables for primary care. It is reported that excellent performance was achieved using non-laboratory with or without laboratory variables (AUROC greater than 0.80) in a diabetes prediction model based on the gradient boosting machine (GBM) algorithm (42) and in hypertension risk prediction models based on XGBoost (43). These findings are consistent with our results, implying great performances in insulin sensitivity and insulin resistance-related disease prediction models developed by machine learning using non-laboratory with or without laboratory features. In our study, non-laboratory variables mainly including WC, BMI, WHtR, RPR, etc., and laboratory variables comprising lipid profile, liver enzymes, etc., are well-known risk factors of low insulin sensitivity (18, 22), which may be one of the important explanations for the eminent performances of our models for the insulin sensitivity assessment developed for the community setting using non-laboratory features and established for primary care using non-laboratory combined with laboratory variables. Lee et al. develop an IR prediction model for a population with chronic kidney disease (44). Tsai et al. train a predictive model for IR in the non-diabetic populations (41). Park et al. develop an insulin resistance index model in the Ansan/Ansung cohort with metabolic diseases were excluded (40). In our present study, we tried to develop predicting models based on machine learning-augmented algorithm in general population in China for insulin sensitivity assessment in the community setting using non-laboratory features and in the primary care setting using non-laboratory combined with easily-obtained laboratory variables.

Our results indicated that the inclusion of laboratory variables in the prediction models significantly improved the performances compared to the models developed exclusively with non-laboratory variables, suggesting the considerable role of laboratory variables in the insulin sensitivity assessment models developed by machine learning. These results seemed consistent with the findings in previous research that the inclusion of laboratory variables such as urinary glucose, urinary vitamin C, and FPG improves the accuracy of diabetes prediction models developed by GBM or LightGBM (42, 45). Noteworthy, the laboratory features (e.g., fasting glucose, serum lipids, liver enzymes, etc.) included in our study were all routinely accessible in primary care, and the predictive models developed with non-laboratory variables can be used instead as a primary screening approach in case that these laboratory features mentioned above are not available.

Additionally, our results showed that the streamlined LightGBM models utilizing the top-20 ranked variables exhibited comparable performances to the models constructed with all features. Likewise, our previous study indicated that the streamlined diabetes prediction model utilizing the top 20 variables developed by the LightGBM algorithm, exhibited great performances similar to that of the model using all variables (45), which is consistent with the findings in the present study. Moreover, it is reported that the simplified model for gestational diabetes prediction using 9 variables based on LightGBM demonstrates only a modest reduction in predictive accuracy compared to its full variable model (38). Importantly, these top 20 variables involved in our simplified model including FPG, WC, BMI, WHtR, TG, and GENDER, etc. are readily obtainable in the community and primary care settings, rendering the simplified model highly convenient and applicable in these scenarios.

Our findings suggested that the LightGBM model demonstrates extraordinary discriminative capability and calibration in the general population, as well as in the male and female sub-populations. Interestingly, the models developed for the male population exhibited slightly better performances than those for the female population, suggesting that it seems necessary to develop relatively specific models for targeted populations to improve the predictive efficacy of the model. The reason for these variations seems unclear and further research is necessary, although they might be relevant to the differences in sex hormone levels, fat distribution (46), etc., between males and females.

The SHAP analysis revealed that features such as obesity, adult weight gain, less exercise, impaired glucose tolerance, hypertriglyceridemia, hypertension, TG/HDL-C ratio, and snoring, were strongly associated with an increased risk of low insulin sensitivity, which is consistent with the findings of previous studies analyzed using traditional statistical methods (18, 20, 47–49). Moreover, our SHAP analysis showed that the number of daughters born, ALT/AST, creatinine, and RPR were risk factors for low insulin sensitivity. It is reported that female fetuses are associated with a higher risk of maternal IR during pregnancy (50), which might be an important explanation for the increased risk of low sensitivity in women who gave birth to more daughters in our study. Anyhow, the detailed mechanism needs to be further investigated. Additionally, it is illustrated that the ALT/AST ratio was the most reliable surrogate measurement for IR in Japanese non-obese people (22), which is consistent with that ALT/AST was a risk factor of low insulin sensitivity in our study. Furthermore, Niu, Y. and colleagues reported that an increase in creatinine elevates the risk of NAFLD (51), which was consistent with the findings of our study since NAFLD is closely related to IR. Moreover, it is demonstrated that resting heart rate was independently associated with first-phase insulin secretion and negatively associated with insulin sensitivity in a non-diabetic population (52), which might be an important explanation that RPR was a risk factor for low insulin sensitivity in the present study. These findings suggest that these features are of utmost importance in the prediction of low insulin sensitivity, and more attention should be paid to them in the prevention and management of IR-related diseases.

Remarkably, our results indicated that personalized predictors could be identified in selected samples, suggesting that ML models combined with SHAP analysis can help screen out key predictors of low insulin sensitivity for specific individuals, and subsequently provide early warning information to get personalized health advice and take more precise measures to alleviate risk of low insulin sensitivity and/or prevent IR-related diseases for them.

Noteworthy, the majority of the features (FPG, WC, BMI, WHtR, etc.) used in the ML models were regularly collected in clinical practice in China, and those features adopted in the ML models which were not routinely collected by medical practitioners (the number of daughters born, number of sons born, education, etc.), can be promptly obtained through easy-to-use electronic questionnaires or open-access web pages. Moreover, the ML models could be further developed and presented in open and accessible web pages to make them easier and more available for residents in communities or clinical practitioners in the setting of primary care to evaluate the risk of low insulin sensitivity, and the information increasingly inputted in the ML model would be extremely helpful to improve the performance of the prediction models in turn, which is one of the biggest advantages of ML (45).

However, it should be noted that although HOMA-IR is commonly used as a surrogate indicator for insulin sensitivity assessment in clinical and epidemiological studies (16), we were unable to use the gold standard - the hyperinsulinemic-euglycemic clamp, to determine insulin sensitivity in this study, so the findings of the current study should be interpreted with caution and further research would be necessary. Additionally, we have not yet explored the predictive efficiency of the model in prospective research, and we plan to perform it in a follow-up study in the near future. Furthermore, the models were developed using data from only one study center, and the participants were over 40 years old. Actually, OGTT-derived methods could be very helpful in the assessment of insulin sensitivity, especially in combination with the determination of plasma glucose and insulin levels using multiple blood samplings and potentially more efficient if they could be integrated in ML models. Regrettably, we were not able to perform OGTT with multiple time points for some practical reasons. Anyhow, we hope that we could do it in the further research in the near future. Moreover, the data used in the present study were obtained from the Han Chinese population in Hubei Province in central China, and herein the generalizability of our models needs further testing with data from more regions and ethnic groups. We would try to develop these ML models into user-friendly web pages or applications that are accessible to the general public and primary care providers, getting more input information and feedback to optimize our models, which is virtually a significant advantage of ML (53). Furthermore, the sensitivity is not as good as the specificity in our LightGBM models, which might be attributed to methodological reasons such as the variables included and/or the algorithms used. Although seven ML algorithms were employed in the present study, there may be other algorithms with better performance available currently or to be developed. It would be necessary for us to further iterate models with more promising algorithms to improve the predictive performance of the models in the future.

Notwithstanding these limitations, the ML models using the LightGBM algorithm, are efficient in predicting insulin sensitivity in the community and primary care settings accurately. Thus, we tentatively put forward that the ML-augmented algorithm might potentially become an efficient and practical tool for insulin sensitivity assessment in community and primary care settings.

## Data availability statement

The original contributions presented in the study are included in the article/**Supplementary Material**. Further inquiries can be directed to the corresponding authors.

## Ethics statement

The studies involving humans were approved by Ethics Committee of Tongji Medical College, Huazhong University of Science and Technology. The studies were conducted in accordance with the local legislation and institutional requirements. The participants provided their written informed consent to participate in this study. Written informed consent was obtained from the individual(s) for the publication of any potentially identifiable images or data included in this article.

## Author contributions

HZ: Methodology, Writing – original draft, Conceptualization, Data curation, Formal analysis, Investigation, Project administration, Supervision, Writing – review & editing. TZ: Data curation, Formal analysis, Investigation, Supervision, Writing – review & editing. JYZ: Data curation, Formal analysis, Investigation, Supervision, Writing – review & editing. JZ: Data curation, Formal analysis, Investigation, Supervision, Writing – review & editing. JM: Conceptualization, Data curation, Formal analysis, Investigation, Supervision, Writing – review & editing. MP: Data curation, Investigation, Supervision, Writing – review & editing. GL: Data curation, Formal analysis, Investigation, Supervision, Writing – review & editing. XZ: Data curation, Formal analysis, Supervision, Writing – review & editing. YW: Investigation, Project administration, Resources, Writing – review & editing. KQ: Investigation, Project administration, Resources, Writing – review & editing. ST: Formal analysis, Investigation, Resources, Writing – review & editing. XL: Investigation, Methodology, Supervision, Validation, Writing – review & editing. HH: Project administration, Resources, Writing – review & editing. MS: Methodology, Supervision, Validation, Writing – review & editing. PW: Conceptualization, Methodology, Supervision, Validation, Writing – review & editing. XH: Conceptualization, Data curation, Investigation, Methodology, Project administration, Supervision, Validation, Writing – original draft, Writing – review & editing. LC: Conceptualization, Data curation, Funding acquisition, Investigation, Methodology, Project administration, Resources, Supervision, Writing – original draft, Writing – review & editing.
