# Supplementary material for: Development and validation of machine learning-augmented algorithm for insulin sensitivity assessment in the community and primary care settings: a population-based study in China
Source: Front Endocrinol (Lausanne). 2024 Jan 25;15:1292346. doi: 10.3389/fendo.2024.1292346 (PMC10850228; doi:10.3389/fendo.2024.1292346)
Supplement: Supplementary file 1 [file Table_1.docx]

**Supplementary materials**

|  | Library | Hyperparameter |
| --- | --- | --- |
| LR | sklearn.linear_model.LogisticRegression (https://scikit-learn.org/stable/modules/generated/sklearn.linear_model.LogisticRegression.html) | 'C': 1, 'penalty': 'l2', 'random_state': 101, 'solver': 'liblinear' |
| SVM | sklearn.svm.SVC (https://scikit-learn.org/stable/modules/generated/sklearn.svm.SVC.html) | "C": 1,  "kernel": "rbf",  "degree": 3,  "gamma": "auto" |
| RF | sklearn.ensemble.RandomForestClassifier (https://scikit-learn.org/stable/modules/generated/sklearn.ensemble.RandomForestClassifier.html) | 'criterion': 'entropy', 'max_depth': 30, 'min_samples_leaf': 1, 'min_samples_split': 2, 'n_estimators': 200, 'random_state': 101 |
| ExtraTrees | sklearn.ensemble.ExtraTreesClassifier (https://scikit-learn.org/stable/modules/generated/sklearn.ensemble.ExtraTreesClassifier.html) | 'criterion': 'gini', 'max_depth': 30, 'min_samples_leaf': 1, 'min_samples_split': 2, 'n_estimators': 200, 'random_state': 101 |
| LightGBM | LightGBM (https://github.com/microsoft/LightGBM) | 'learning_rate': 0.1, 'max_depth': 20, 'n_estimators': 100, 'num_leaves': 21, 'reg_alpha': 0, 'reg_lambda': 0, 'subsample': 0.8 |
| XGBoost | XGBoost (https://xgboost.readthedocs.io/en/stable/python/python_api.html#) | 'gamma': 0.5, 'learning_rate': 0.1, 'max_depth': 9, 'min_child_weight': 5, 'reg_alpha': 0.1, 'reg_lambda': 0, 'subsample': 0.8 |
| CART | sklearn.tree.DecisionTreeClassifier (https://scikit-learn.org/stable/modules/generated/sklearn.tree.DecisionTreeClassifier.html) | 'criterion': 'entropy', 'max_depth': 9, 'min_samples_leaf': 6, 'min_samples_split': 2 |

**Supplementary Table 1. The hyperparameter in the models with seven ML algorithms in community settings**

|  | Library | Hyperparameter |
| --- | --- | --- |
| LR | sklearn.linear_model.LogisticRegression (https://scikit-learn.org/stable/modules/generated/sklearn.linear_model.LogisticRegression.html) | 'C': 1, 'penalty': 'l2', 'random_state': 101, 'solver': 'liblinear' |
| SVM | sklearn.svm.SVC (https://scikit-learn.org/stable/modules/generated/sklearn.svm.SVC.html) | "C": 1,  "kernel": "rbf",  "degree": 3,  "gamma": "auto" |
| RF | sklearn.ensemble.RandomForestClassifier (https://scikit-learn.org/stable/modules/generated/sklearn.ensemble.RandomForestClassifier.html) | 'criterion': 'entropy', 'max_depth': 30, 'min_samples_leaf': 1, 'min_samples_split': 2, 'n_estimators': 200, 'random_state': 101 |
| ExtraTrees | sklearn.ensemble.ExtraTreesClassifier (https://scikit-learn.org/stable/modules/generated/sklearn.ensemble.ExtraTreesClassifier.html) | 'criterion': 'entropy', 'max_depth': 30, 'min_samples_leaf': 1, 'min_samples_split': 2, 'n_estimators': 200, 'random_state': 101 |
| LightGBM | LightGBM (https://github.com/microsoft/LightGBM) | 'learning_rate': 0.2, 'max_depth': 20, 'n_estimators': 500, 'num_leaves': 41, 'reg_alpha': 0.05, 'reg_lambda': 0.1, 'subsample': 0.8 |
| XGBoost | XGBoost (https://xgboost.readthedocs.io/en/stable/python/python_api.html#) | 'gamma': 0.1, 'learning_rate': 0.1, 'max_depth': 9, 'min_child_weight': 1, 'reg_alpha': 0.01, 'reg_lambda': 0.1, 'subsample': 0.8 |
| CART | sklearn.tree.DecisionTreeClassifier (https://scikit-learn.org/stable/modules/generated/sklearn.tree.DecisionTreeClassifier.html) | 'criterion': 'entropy', 'max_depth': 9, 'min_samples_leaf': 6, 'min_samples_split': 2 |

**Supplementary Table 2. The hyperparameter in the models with seven ML algorithms in primary care settings**

**Abbreviation:** ML, machine learning; LR, Logistic Regression; SVM, Support Vector Machine; RF, Random Forest; ExtraTrees, Extremely randomized trees; LightGBM, Light Gradient Boosting Machine; XGBoost, eXtreme Gradient Boosting; CART, Classification and Regression Tree.
